# Facial appearance and metabolic health biomarkers in women

**DOI:** 10.1038/s41598-020-70119-6

**Published:** 2020-08-03

**Authors:** Agnieszka Żelaźniewicz, Judyta Nowak, Patrycja Łącka, Bogusław Pawłowski

**Affiliations:** Department of Human Biology, University of Wrocław, ul. Kuźnicza 35, 50-138 Wrocław, Poland

**Keywords:** Evolution, Physiology, Psychology

## Abstract

Facial appearance has been suggested to provide an honest cue of an individual’s biological condition. However, there is little direct evidence that facial attractiveness reflects actual health. Here we tested if facial appearance is related with metabolic health biomarkers. Face photographs of 161 healthy, young women (M_age_ = 28.59, SD_age_ = 2.34) were assessed in terms of perceived attractiveness and health. Metabolic health was evaluated based on levels of markers of lipid and glucose metabolism balance, liver functioning, and inflammation. BMI, testosterone (T), and estradiol (E2) levels were controlled. Facial attractiveness, but not health, was negatively related with lipid profile components detrimental to health (total cholesterol, LDL, triglycerides) but not with relatively protective for health HDL. When controlled for BMI, E2, and T, only the relationship between attractiveness and triglycerides remained significant. Facial appearance was unrelated with glucose metabolism, liver functioning, and inflammatory markers. The results suggest, that for healthy women of reproductive age, such measures as BMI and sex hormone levels may be better predictors of attractiveness, compared to measures of metabolic health. Markers of lipid, glucose homeostasis, liver functioning or low-grade inflammation may be rather indicators of future health, of lesser importance in mating context, thus only modestly reflected in facial appearance.

## Introduction

Evolutionary theories propose that facial attractiveness is a cue of an individual’s biological condition^1,2^. Thus, facial attractiveness and individual characteristics, cross-culturally perceived as attractive, such as sexual dimorphism^3,4^, symmetry^5^, averageness^6^, carotenoid-linked skin colour^7^, and homogenous skin texture^8,9^ are expected to develop and be displayed only in individuals with good health and high fertility, and to correlate with various measures of biological condition^10,11^, allowing for the best possible partner selection.

Although perceived facial attractiveness is widely recognized as such honest cue of an individual’s health^11–16^, there are relatively few studies testing this assumption directly. Furthermore, the results of these studies are ambiguous, indicating none^17^ or positive correlation between perceived attractiveness and health measures, however inconsistent across sexes or the studied measures^7,18^. Male facial attractiveness has been suggested to reflect the strength of antibody response to vaccination^19^ and cytokine response after stimulation with lipopolysaccharide^20^, two direct measures of immune functioning. Furthermore, Gangestad et al.^21^ showed that male facial attractiveness, symmetry and masculinity are negatively related with oxidative stress level, an important physiological measure of general body functioning, underlying many pathological processes and diseases. For women, the results are more ambiguous and, in fact, there is little direct evidence that female facial appearance signals immunity or health. Some research has shown that women’s facial attractiveness correlates negatively with cortisol level and percentage of body fat but not with the strength of antibody response to vaccination^17^ or salivary immunoglobulin A level^22^. Also, Foo et al.^7^ showed that neither attractiveness nor its components (facial femininity and adiposity) were related to immune functioning or oxidative stress level in women. However, other studies have suggested that women’s facial femininity reflects better physical health and fewer infections per year^23^ or fewer past health problems^18,24,25^.

Most of the research in this area is characterized by relatively infrequent use of non-student samples^26^ and focus on health markers related mainly with innate or acquired immunity^17,19,20^, self-reported infection frequency^18,23,25^, sex hormones levels^3^ or oxidative balance^21^, neglecting other important factors, underlying current and future health, such as metabolic homeostasis, that may also be reflected in physical appearance and shape preferences in mate choice context. From evolutionary point of view, health can be understood as an organism’s optimal functioning, resulting not only from many physiological processes and body functions, but also from biological trade-offs between them, occurring throughout the life course, assuring maximal possible fitness in given genetic and environmental conditions^27,28^. As such, it may be difficult to reliably evaluate an individual’s biological condition, based on a single health component, and studies on the relationship between physical attractiveness and other than immunity health components may help to resolve discrepancies in previous results.

So far, no study has examined the relationship between facial appearance and markers of metabolic health, underlying many current and future health problems and related with the crucial components of an individual’s biological condition (e.g. immunity and/or fertility). Lipid profile is strongly linked not only with cardio-metabolic health, mortality risk, but also with fertility^29^, and as such, may predict women’s reproductive success. For instance, cholesterol is a substrate for estradiol and progesterone synthesis^30^, directly related with female fecundity^31^. Furthermore, maternal cholesterol metabolism during pregnancy is crucial for fetus development and has a long-term impact on offspring’s adult health^32^. Also, glucose metabolism may be reflected in facial appearance, not only as a major metabolic determinant of the ageing process^33^ and longevity^34–36^, but also due to its relationship with skin ageing^37,38^, and perceived age^39^. Furthermore, liver malfunction may be quickly reflected in facial appearance, as it can result in various cutaneous and mucosal changes, as well as in pruritus and abnormal hair growth, that can be easily seen with the naked eye^40,41^. Also, chronic low-grade inflammation, i.e. a minor elevation in the baseline concentration of inflammatory markers such as C-reactive protein (CRP) or interleukin 6 (IL-6), is recognized as an important factor, underlying many common diseases^42,43^, fertility problems^44^, and ageing processes^45^.

Here, we tested if a woman’s facial appearance is a cue to her metabolic health. In a sample of non-student women of reproductive age, we investigated the relationship between facial attractiveness and: (1) serum lipid profile (total cholesterol, high density lipoprotein (HDL), low density lipoprotein (LDL), triglycerides levels), (2) glucose homeostasis markers [glycated hemoglobin (HbA1C), HOMA-IR score (homeostatic model assessment for insulin resistance), the connecting peptide (C-peptide)], (3) liver enzymes [alanine aminotransferase (ALT), aspartate aminotransferase (AST)], and (4) non-specific inflammation markers [high sensitivity CRP (hsCRP), IL-6]. We hypothesized that facial attractiveness is negatively related to the levels of proatherogenic cholesterol, triglycerides and LDL and positively related to antiatherogenic HDL. We hypothesized, that levels of such measures of glucose homeostasis as HbA1C, HOMAI-IR, and C-peptide negatively predict women’s facial attractiveness. Furthermore, we hypothesized that liver enzymes, hsCRP and IL-6 levels are negatively related with facial attractiveness. Additionally, as estradiol^3^ and testosterone^46^ may impact not only a woman’s facial attractiveness, but also levels of health biomarkers used in this study^47–50^, we included these hormones in the analyses. In order to control for the possible impact of menstrual cycle on metabolic health markers levels^51–53^, women were invited to participate in the study in the early follicular phase, recommended for evaluation of metabolic markers among reproductive-aged women^54,55^. We also controlled for age and BMI due to their potential impact on women’s facial attractiveness^56,57^ and metabolic health biomarkers^58^. Finally, we verified if all above mentioned measures of health are reflected not only in perceived facial attractiveness but also in perceived facial health.

## Material and methods

This research was a part of a broader project on women’s health, conducted on 211 women (M_age_ = 28.51, SD_age_ = 2.37) from a western, urban population. Participants were recruited by information in local newspapers, radio, social networks and advertisement. The protocol used to recruit participants and collect data was approved by local ethics committee. The general purpose of the study was explained and informed, written consent was obtained for participation in the study and use of data for scientific purposes from all participants.

### Participants and general procedure

Face photographs and complete results of blood analysis were available for 171 women between 24 and 34 years old (M_age_ = 28.61, SD_age_ = 2.34), constituting the study sample for this research. The criteria for inclusion were: no fertility problems (including PCOS), never been pregnant (including miscarriages), no hormonal contraception or medications in the six months prior to study participation, no chronic diseases (diabetes, thyroid problems, etc.), and no acute infections (cold, flu, tooth extraction, etc.). Women’s health status was verified based on medical control, blood morphology with smear and CRP level. All women were asked to contact the research team at the 1st day of the menstrual cycle (self-assessed) and were invited to participate in the study between the 2nd and the 4th day of menstrual cycle (early follicular phase). Ten participants were excluded from the analyses due to the following reasons: (a) elevated levels of inflammation biomarkers (CRP > 10 µg/ml), indicating ongoing inflammatory state (N = 4); (b) inadequate menstrual cycle day (N = 5); (c) testosterone level exceeding three standard deviation (N = 1). The final analyses included 161 women (M_age_ = 28.59, SD_age_ = 2.34).

During the visit, a fasting blood sample was taken between 7:30 and 9:00 a.m. for further blood biochemical and hormonal analyses. Participants were weighed, measured and BMI was calculated. Photos of women’s faces were taken. Participants also filled personal questionnaire, containing questions on date of birth, education level, and also, in order to verify their health status, questions on past and current health problems and medication use.

### Biochemical analyses of health biomarkers

Biochemical analyses of blood health markers included evaluation of: (1) lipid profile (total cholesterol, HDL, LDL, triglycerides); (2) glucose homeostasis markers (HbA1C), HOMA-IR index [calculated based on fasting glucose and insulin levels, according to the formula: insulin (mU/ml) × glucose (mmol/l)/22.5] and C-peptide level; (3) liver enzymes [alanine transaminase (ALT) and aspartate transaminase (AST)]; (4) inflammatory markers (hsCRP, hsIL-6). All measures, except inflammation markers were measured in professional analytical laboratory.

HsCRP and hsIL-6 were measured by immunoassay and commercial ELISA kit (DEMEDITEC cat. no. DE740011, and R&D cat. no. HS600B). Inter- and intra-assay precision, provided by the manufacturer, were < 6.3% , < 6.9% for hsCRP and < 9.6%, < 7.8% for hsIL-6. Assay sensitivity was 0.02 µg/ml for hsCRP and 0.039 pg/ml for hsIL-6. Sample and reagents preparation as well as assay procedures were carried out in accordance to the manufacturers’ instructions. Samples were assayed in duplicate and the average absorbance value was used to calculate hormone concentration. Standard curves were created by plotting the mean absorbance value (Y axis) for each standard against its concentration (X axis). The best fit line was used for calculation the individual's levels of hsCRP or hsIL-6 in each sample. The concentrations were expressed in µg/ml for hsCRP and pg/ml for hsIL-6.

### Hormones levels analyses

Estradiol (E2) level was assayed in certified laboratory (DIAGNOSTYKA) using ElectroChemi Luminescence immunoassay and Cobas analyzer (Roche Diagnostic) and expressed in pg/ml.

Quantitative measurement of total testosterone (tT) were determined by enzyme immunoassay using commercial kits (DEMEDITEC, cat. no. DE1559). Inter- and intra-assay coefficients of variation, provided by the manufacturer, were < 10% and < 4.2% respectively, with a test sensitivity 0.08 ng/mL. Assay procedure was performed in accordance the manufacturer’s instruction. Samples were assayed in duplicate and the average absorbance value was used to calculate hormone concentration. Standard curve was created by plotting absorbance values for each standard (Y axis) against its concentration (X axis). Total testosterone concentration was calculated in relation to standard curve and expressed in ng/ml.

### Stimuli and on-line survey

Face photographs of all participants were taken with the same digital still camera (Nikon D7100 with Tamron SP AF 17-50 mm F/2.8 XR Di II LD IF camera lens) at a distance of 2.0 m, using the same general camera setting and constant lighting conditions. Participants were seated on a fixed chair and the height of the camera was adjusted to a woman’s height. Women had no make-up (participants were asked to come to the lab without make-up or had a possibility to remove their make-up at the lab), were asked to pose with a neutral facial expression, to remove glasses or earrings, and to wear a hairband (to make sure that face was fully visible). The background was replaced with a uniform white colour. In the photos, white ovals were placed around the women’s faces to obscure information about hairstyles.

161 photographs were randomly divided into eleven on-line surveys. Each survey contained up to 15 photographs, in order to avoid presenting too many stimuli to one respondent, what might cause fatigue and impact credibility of the assessment. Photographs were presented in random order. Based on facial appearance, men were asked to evaluate women’s attractiveness (“How attractive is the woman in the photo”) and health (“How healthy is the woman in the photo”) on the scale from 1 (not at all attractive/unhealthy) to 9 (very attractive/healthy). Each photo was rated by 100 men aged 18–39 years (M_age_ = 23.74, SD_age_ = 4.26), recruited on-line, via social media, internet forums, and university webpages. Informed consent was obtained for participation in the study from all participants. Inter-rater agreements for attractiveness and health were high (attractiveness: Cronbach’s α = 0.98; health: Cronbach’s α = 0.95) and mean attractiveness and health ratings were used in the statistical analyses.

### Statistical methods

As values of health markers, E2 and tT levels were not distributed normally across participants, logarithmic values were used in the analyses. First, to explore the data, we performed zero-order correlation analyses between facial appearance and health markers (cholesterol, HDL, LDL, triglycerides, ALT, AST, HbA1C, HOMA-IR, C-peptide, hsCRP, IL-6 levels) and also controlled variables (BMI, age, tT, and E2). Benjamini–Hochberg adjustment^59^ was used to correct *p*-values for multiple testing. Additionally, we also run zero-order correlations between controlled variables and health biomarkers. As age was not related with facial appearance we did not include age in further analyses.

Although facial appearance ratings were nested in the surveys (the photos were divided between the surveys and rated by different sets of men), preliminary analyses (nested Mixed Model), showed the Intraclass Correlation Coefficient to be equal to 0, indicating that data were not hierarchical, and that unbiased estimates can be obtained from ordinary multiple regression.

In order to reduce the number of predictors and to summarize the interrelated health markers variables we conducted Principal Component Analysis on metabolic health markers (total cholesterol, HDL, LDL, triglycerides, HbA1C, HOMA-IR, C-peptide, ALT, AST, hsCRP, and Il-6 leves). After obtaining principal components (data reduction in Supplementary Materials—Table S3), we ran two multiple regressions with facial attractiveness and perceived health as dependent variables, and principal components as predictors, including BMI and sex steroids levels as controlled variables.

Analyses were performed with Statistica 12.0 software. The results were interpreted as statistically significant if *p* < 0.05.

### Ethics statement

The research was approved by the by Ethics Committee of Lower Silesian Chamber of Physicians (2/BO/2016). The general purpose of the study was explained and written consent was obtained for participation in the study and use of data for scientific purposes from all participants. All medical procedures, including participants examination and blood collection, have been conducted by certified medical staff at the medical clinic. All procedure was consistent with the guideline included in the "Declaration of Helsinki—Ethical Principles for Medical Research Involving Human Subjects" formulated by World Medical Association in 2013 (https://www.wma.net/policies-post/wma-declaration-of-helsinki-ethical-principles-for-medical-research-involving-human-subjects/).

## Results

### Descriptive statistics

Mean values, ranges and standard deviations of facial attractiveness and facial health assessment, health markers, age and controlled variables are presented in Table 1. Although facial attractiveness and health assessment were strongly correlated (r = 0.78, *p* < 0.001, 95% CI [0.71; 0.83]) test for dependent samples showed that men tended to give higher facial health ratings (M = 4.80 ± 0.74) compared with facial attractiveness assessment (M = 3.80 ± 0.98) (t(160) = − 20.96, *p* < 0.001).

**Table 1:** Descriptive statistics of the analysed variables (N = 161).

	M	SD	Min	Max
Age (year)	28.59	2.34	25.00	33.00
Total cholesterol (mg/dl)	178.30	26.48	117.00	242.00
HDL (mg/dl)	71.79	15.34	36.00	108.00
LDL (mg/dl)	91.71	22.71	32.00	155.00
Triglycerides (mg/dl)	74.04	33.03	32.00	270.00
HbA1C (%)	5.04	0.20	4.40	5.50
HOMA-IR	1.52	0.81	0.46	5.27
C-peptide (ng/ml)	1.61	0.49	0.74	3.86
ALT (U/l)	14.23	6.58	6.00	60.00
AST (U/l)	19.01	11.01	10.00	143.00
hsCRP (µg/ml)	1.11	1.36	0.003	7.06
IL-6 (pg/ml)	1.04	0.96	0.08	6.92
Face attractiveness (1–9)	3.80	0.98	1.82	6.32
Perceived health (1–9)	4.81	0.74	3.17	7.45
BMI (kg/m^2^)	22.08	3.52	16.34	35.40
Estradiol (pg/ml)	35.55	17.69	5.00	110.00
tTestosterone (ng/ml)	0.53	0.21	0.30	1.85

Zero-order correlation analyses for the relationship between health biomarkers and controlled variables are presented in Supplementary Material.

### Perceived facial attractiveness and health & health biomarkers

Zero-order correlation analyses revealed that facial attractiveness correlated negatively with total cholesterol, LDL, triglycerides, BMI, and tT level and positively with E2 level. However, the correlations between facial attractiveness and total cholesterol and tT were non-significant when corrected for multiple comparisons (Supplementary Material, Table S2). There was no correlation between facial attractiveness and HDL, glucose homeostasis, markers of liver functioning, inflammatory markers. Perceived facial health was not related with lipid profile, glucose homeostasis or markers of liver functioning. There was a negative relationship between perceived health and hsCRP level, BMI and tT level and positive with E2 level (Table 2). However, the relationship between perceived health and hsCRP was not significant when *p-*value was corrected for multiple comparisons (Supplementary Materials, Table S2).

**Table 2:** Correlation values for the relationship between biomarkers of health and face attractiveness and health assessment, with the corresponding *p-values* (N = 161).

	Face attractiveness			Perceived health		
	r	*p*	%95 CI	r	*p*	%95 CI
LOG total cholesterol (mg/dl)	− **0.18**	**0.02**	[− 0.32; − 0.03]	− 0.08	0.28	[− 0.23; 0.07]
LOG HDL (mg/dl)	0.11	0.16	[− 0.04; 0.26]	0.06	0.44	[− 0.09; 0.21]
LOG LDL (mg/dl)	**− 0.21**	**0.009**	[− 0.35; − 0.06]	− 0.11	0.15	[− 0.26; 0.04]
LOG triglycerides (mg/dl)	**− 0.25**	**0.002**	[− 0.39; − 0.10]	− 0.08	0.30	[− 0.23; 0.07]
LOG HbA1C (%)	− 0.10	0.20	[− 0.25; 0.05]	− 0.05	0.49	[− 0.20; 0.10]
LOG HOMA-IR	− 0.10	0.21	[− 0.25; 0.05]	− 0.12	0.13	[− 0.27; 0.03]
LOG C-peptide (ng/ml)	− 0.05	0.55	[− 0.20; 0.10]	0.02	0.76	[− 0.13; 0.17]
LOG ALT (U/l)	− 0.06	0.43	[− 0.21; 0.09]	− 0.12	0.12	[− 0.27; 0.03]
LOG AST (U/l)	0.02	0.78	[− 0.13; 0.17]	− 0.04	0.60	[− 0.19; 0.11]
LOG hsCRP (µg/ml)	− 0.14	0.08	[− 0.29; 0.01]	**− 0.18**	**0.02**	[− 0.32; − 0.03]
LOG hsIL-6 (pg/ml)	− 0.10	0.19	[− 0.25; 0.05]	− 0.09	0.24	[− 0.24; 0.06]
BMI (kg/m^2^)	**− 0.26**	** < 0.001**	[− 0.40; − 0.11]	**− 0.22**	**0.004**	[− 0.36; − 0.07]
Age (year)	− 0.03	0.67	[− 0.18; 0.12]	0.08	0.31	[− 0.07; 0.23]
LOG tTestosterone (ng/ml)	**− 0.16**	**0.04**	[− 0.31; − 0.01]	**− 0.22**	**0.006**	[− 0.36; − 0.07]
LOG E2 (pg/ml)	**0.20**	**0.01**	[0.05; 0.34]	**0.24**	**0.002**	[0.09; 0.40]

Bolded values are significant at *p* < 0.05.

Principal component analysis returned four PCs (see supplementary material for details on data reduction—Table S3). PCA returned four PCs with eigenvalues > 1. PC1 was loaded mainly by glucose homeostasis markers (HOMA-IR, C-peptide). PC2 was loaded mainly by lipid profile, mainly markers negatively predicting health (total cholesterol, LDL and triglycerides level). PC3 was loaded mainly by liver enzymes (ALT, AST). PC4 was loaded mainly by inflammatory markers (hsCRP, Il-6). The four PCs jointly explained 69.82% of the variation in health markers variables.

Regression analysis showed that facial attractiveness was mainly predicted by PC2, but unrelated with PC1, PC3 or PC4, when controlled for BMI and sex steroids levels (Table 3).

**Table 3:** Results of regression analyses for the relationship between facial attractiveness and four PCs controlled for covariates (F(7,153) = 3.92, *adj.* R^2^ = 0.11, *p* < 0.001, Cohen’s f^f^ = 0.18; N = 161).

	β	SE (β)	t(153)	*p*
PC1	− 0.001	0.09	− 0.01	0.99
PC2	**− 0.17**	**0.07**	**− 2.24**	**0.03**
PC3	0.06	0.08	0.81	0.42
PC4	− 0.10	0.08	− 1.31	0.19
BMI	− 0.17	0.10	− 1.80	0.07
LOG E2	0.14	0.08	1.86	0.06
LOG tT	− 0.14	0.07	− 1.89	0.06

Bolded values are significant at *p* < 0.05.

We found no relationship between perceived health and any of the PCs, when controlled for BMI and sex steroids levels. Perceived health was only predicted by sex steroids levels (Table 4).

**Table 4:** Results of regression analyses for the relationship between facial attractiveness and four PCs controlled for covariates (F(7,153) = 3.70, *adj.* R^2^ = 0.10, Cohen’s f^f^ = 0.16; *p* = 0.001, observed power N = 161).

	Β	SE (β)	t(153)	*p*
PC1	0.06	0.09	0.63	0.53
PC2	− 0.07	0.08	− 0.89	0.38
PC3	− 0.01	0.08	− 0.20	0.84
PC4	− 0.10	0.08	− 1.22	0.22
BMI	− 0.15	0.10	− 1.58	0.12
LOG E2	**0.18**	**0.08**	**2.36**	**0.02**
LOG tT	**− 0.20**	**0.08**	**− 2.60**	**0.01**

Bolded values are significant at *p* < 0.05.

## Discussion

The results of our study showed that facial appearance in healthy women of reproductive age was predicted by lipid profile components, but unrelated with glucose metabolism markers, liver enzyme, and low-grade inflammation markers levels. The results were similar also when controlled for BMI and sex hormones levels. Among lipid parameters, facial attractiveness, but not perceived health, was negatively related with triglycerides, LDL, and total cholesterol (although the latter relationship was not significant when *p* was adjusted for multiple testing), but not HDL level. Additionally, we confirmed the negative relationship between facial appearance (attractiveness and health) and BMI and testosterone levels (although the relationship was not significant when *p* was adjusted for multiple testing) and positive with estradiol level, reported in previous research^3,46,56,57^.

Distinct lipid profile components are differently related with health risks^60,61^ and women’s reproductive functions^62^. Cholesterol, LDL and triglycerides increase, whereas HDL decreases, the risk of cardiovascular diseases, health problems, and mortality^50,63^. We found that facial attractiveness was predicted by components detrimental for health and unrelated with relatively protective for health HDL. This result aligns with the “avoidance of bad genes” hypothesis^64^. From an adaptive view, it might be more important to be more vigilant to and to avoid cues of compromised health and reduced fitness, than look for a perfectly healthy partner, who may be difficult, or even impossible, to be found. This may drive a stronger negative relationship between attractiveness and compromised health and weaker positive relationship between attractiveness and improved health.

We hypothesized that the relationship between facial appearance and lipid profile may be driven by its impact on current and future health and/or by its role as a substrate for sex hormones synthesis^30^, which have been shown to be related with facial appearance^3,17,46^. However, although we confirmed the relationship between sex steroids and facial appearance, the lipid profile was only related with facial attractiveness and unrelated with facial health, and presumably it was not the level of sex hormones that explained the relationship between facial attractiveness and lipid profile, but rather BMI, negatively related with both, perceived facial attractiveness and health, but also lipid profile markers (whereas sex steroids were not related with lipid profile). Dyslipidaemia is one of the most common health consequences of excessive body adiposity^65^, and thus facial appearance in women with high BMI may be the key morphological cue of imbalanced lipid profile (and also other health problems) linked to excessive adiposity level. Similarly, Phalane et al.^20^ showed that facial attractiveness is a cue to an individual’s health, as it correlates with immune system functioning, and even more strongly with BMI, a common measure of general health, related also with immunity^66^. Possibly, sexual selection mechanisms shaped stronger aversion against morphological cues related with many health and fertility problems (not only dyslipidaemia or lower immunity), such as higher body adiposity, rather than against cues of specific health problems. Thus, the relationship between specific health problems and physical attractiveness may remain weaker, potentially resulting in ambiguous results of the studies, employing only selected, not comprehensive measures of health^7,17,18^, whereas the negative relationship between facial adiposity and perceived attractiveness has been observed cross-culturally^18,56,67^.

We found no relationship between facial appearance and glucose homeostasis or liver functioning parameters. Previous research showed that glucose level is positively related with perceived age in elderly individuals^39^, an important predictor of facial attractiveness in women^9^ but we failed to find similar effect in relatively young and healthy women, that participated in our study. Possibly the relationship between attractiveness and these health markers can only be detected in individuals of older age or when comparing healthy individuals with individuals with serious health problems, resulting in chronic increased glucose levels or liver transaminases (but not comparing individuals within normal range of these health markers). This presumption has been somehow confirmed in the previous study showing no correlation between facial attractiveness and health when teen photos were evaluated, whereas more attractive elderly people were assessed also as healthier than their peers^68^.

Furthermore, although in this research facial attractiveness and health assessments were strongly correlated, the relationships between these two ratings and health biomarkers levels differed. Except for BMI and sex steroids, perceived facial health was only weakly related with hsCRP level (with *p* value non-significant after adjustment for multiple testing). Also, facial health ratings were higher compared with facial attractiveness ratings, possibly indicating that health perception is less evolutionary relevant compared to attractiveness perception, as in mate choice individuals choose potential partners based on rather automatic and more strict/determined aesthetic preference (perceived attractiveness) and not based on rather conscious and less strict/determined assessment of health. However, this presumption is only speculative and would require further studies to verify it.

Some limitations of our study need to be addressed as well. First, metabolic health markers and sex steroid hormones levels were assessed only at the between-subjects level, based on a single measurement. Thus, it would be worth to verify the results of our study with repeated metabolic markers and sex steroids measurements, using a longitudinal, rather than cross-sectional design to assess the relationship between metabolic health markers and a woman’s appearance. Another potential limitation is the self-reported moment of menstrual cycle, not confirmed by an objective test. Although women were instructed to visit the lab in the exact days of menstrual cycle and were repeatedly reminded that the date of the visit can be rescheduled, according to a participant’s menstrual cycle pattern, we cannot exclude the possibility that some participants visited the lab at some other day of the menstrual cycle. Future work would benefit from using repeated hormone tests to confirm the moment of the menstrual cycle.

The results of our study suggest, that for healthy women, at a peak of reproductive age, BMI and sex hormones levels may be better predictors of their biological condition, reflected in the level of physical attractiveness, as they are strongly linked to current health and fecundity^31,69^, although for no relationship between sex steroids and facial attractiveness in women see also^70^^,^ compared to specific measures of metabolic health, such as markers of lipid and glucose homeostasis, liver functioning or low-grade inflammation. Within clinical norm, these measures of health, assessed in relatively young women, without any chronic health problems, may be rather indicators of future health problems, of lesser importance in mating context and only modestly reflected in facial appearance.

## Supplementary information

Supplementary Information.
