# Supplementary material for: Facial appearance and metabolic health biomarkers in women
Source: Sci Rep. 2020 Aug 3;10:13067. doi: 10.1038/s41598-020-70119-6 (PMC7398920; doi:10.1038/s41598-020-70119-6)
Supplement: Supplementary file 1 — Supplementary Information. [file 41598_2020_70119_MOESM1_ESM.docx]

**Title: Facial appearance and metabolic health biomarkers in women.**

*Running head: Facial appearance & health*

Authors: Agnieszka Żelaźniewicz^1*^, Judyta Nowak^1^, Patrycja Łącka^1^, Bogusław Pawłowski^1^

^1^ Department of Human Biology, University of Wrocław

**Funding:**

| This work was supported by National Science Centre, Poland; Grant number 015/19/B/NZ8/02061 |  |
| --- | --- |

**Electronic supplementary material:**

**Fig. S1 Graph, summarizing measures of health and predicted direction of the relationship with facial attractiveness/perceived health assessment ([-] – negative relationship; [+] – positive relationship).**


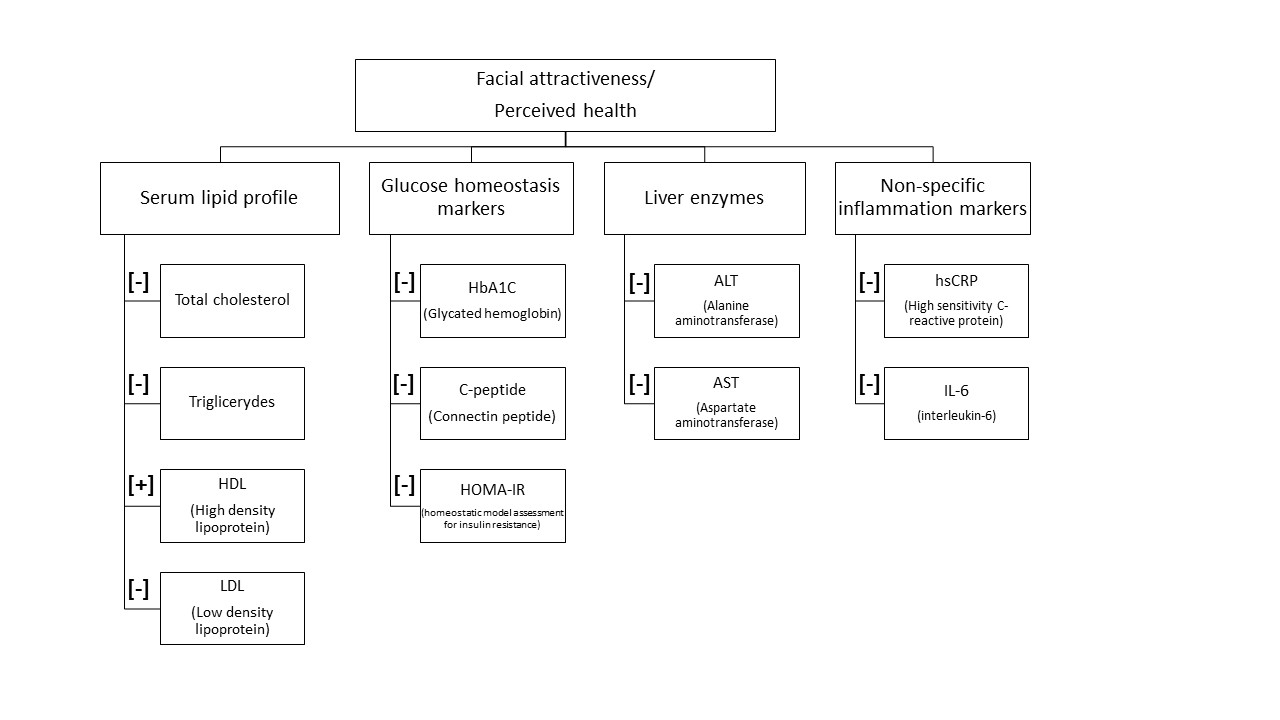


**I DATA EXPLORATION**

Table S1. Zero-order correlation analyses for the relationship between health biomarkers and controlled variables (N = 161). Significant results are bolded.

|  | BMI [kg/m^2^] | | | LOG tTestosterone [ng/ml] | | | LOG E2 [pg/ml] | | |
| --- | --- | --- | --- | --- | --- | --- | --- | --- | --- |
|  | r | *p* | 95% CI | r | *p* | 95% CI | r | *p* | 95% CI |
| LOG Total cholesterol [mg/dl] | 0.12 | 0.13 | [-0.03; 0.27] | -0.02 | 0.75 | [-0.17; 0.13] | -0.04 | 0.59 | [-0.19; 0.11] |
| LOG HDL [mg/dl] | **-0.38** | **<0.001** | **[-0.50; -0.24]** | -0.10 | 0.17 | [-0.25; 0.05] | 0.04 | 0.61 | [-0.11; 0.19] |
| LOG LDL [mg/dl] | **0.25** | **0.001** | **[0.10; 0.39]** | 0.05 | 0.54 | [-0.10; 0.20] | -0.07 | 0.36 | [-0.22; 0.08] |
| LOG Triglycerides [mg/dl] | **0.36** | **<0.001** | **[0.22; 0.49]** | 0.14 | 0.07 | [-0.01; 0.29] | -0.06 | 0.43 | [-0.21; 0.09] |
| LOG HbA1C [%] | 0.09 | 0.26 | [-0.06; 0.24] | 0.02 | 0.76 | [-0.13; 0.17] | **-0.17** | **0.03** | **[-0.31; -0.01]** |
| LOG HOMA-IR | **0.43** | **<0.001** | **[0.29; 0.55]** | 0.11 | 0.15 | [-0.04; 0.26] | 0.02 | 0.84 | [-0.13; 0.17] |
| LOG C-peptid [ng/ml] | **0.44** | **<0.001** | **[0.30; 0.55]** | 0.08 | 0.33 | [-0.07; 0.23] | -0.01 | 0.85 | [-0.16; 0.14] |
| LOG ALT [U/l] | **0.36** | **<0.001** | **[0.22; 0.49]** | **0.18** | **0.02** | **[0.03; 0.32]** | -0.08 | 0.32 | [-0.23; 0.07] |
| LOG AST [U/l] | 0.08 | 0.34 | [-0.07; 0.23] | 0.01 | 0.90 | [-0.14; 0.16] | **-0.17** | **0.03** | **[-0.31; -0.01]** |
| hsCRP [µg/ml] | **0.36** | **<0.001** | **[0.22; 0.49]** | -0.06 | 0.41 | **[-0.21; 0.09]** | -0.14 | 0.08 | [-0.29; 0.01] |
| hsIL-6 [pg/ml] | 0.08 | 0.32 | [-0.07; 0.23] | 0.02 | 0.83 | [-0.13; 0.17] | -0.05 | 0.48 | [-0.20; 0.10] |

Figure S2a. Plot of the relationship of perceived facial attractiveness and E2 (r=0.20, *p*=0.01).

Figure S2b. Plot of the relationship of perceived facial health and E2 (r=0.24, *p*=0.002).

Figure S3a. Plot of the relationship of perceived facial attractiveness and BMI (r=-0.26, *p*<0.001).

Figure S3b. Plot of the relationship of perceived health and BMI (r=-0.22, *p*=0.004).

Figure S3a. Plot of the relationship of perceived facial attractiveness and testosterone (r=-0.18, *p*=0.02).

Figure S3b. Plot of the relationship of perceived health and testosterone level (r=-0.23, *p*=0.003).

**II ADJUSTMENT FOR MULTIPLE TESTING**

Table S2. Correlation values for the relationship between biomarkers of health and face attractiveness and health assessment, with the corresponding *p-values* (N=161). Bolded values are significant after adjustment for multiple testing (i.e. (i/m)Q>*p*)

|  | Face attractiveness | | | | Perceived health | | | |
| --- | --- | --- | --- | --- | --- | --- | --- | --- |
|  | r | *p* | Rank^1^ | (i/m)Q^2^ | r | *p* | Rank^1^ | (i/m)Q^2^ |
| LOG Total Cholesterol [mg/dl] | -0.18 | 0.020 | 8 | 0.013 | -0.08 | 0.284 | 20 | 0.033 |
| LOG HDL [mg/dl] | 0.11 | 0.156 | 15 | 0.025 | 0.06 | 0.440 | 24 | 0.040 |
| LOG LDL [mg/dl] | **-0.21** | **0.009** | **6** | **0.010** | -0.11 | 0.150 | 14 | 0.023 |
| LOG Triglycerides [mg/dl] | **-0.25** | **0.002** | **2** | **0.003** | -0.08 | 0.298 | 21 | 0.035 |
| LOG HbA1C [%] | -0.10 | 0.196 | 17 | 0.028 | -0.05 | 0.492 | 25 | 0.042 |
| LOG HOMA-IR | -0.10 | 0.206 | 18 | 0.030 | -0.12 | 0.126 | 13 | 0.022 |
| LOG C-peptide [ng/ml] | -0.05 | 0.555 | 26 | 0.043 | 0.02 | 0.759 | 29 | 0.048 |
| LOG ALT [U/l] | -0.06 | 0.430 | 23 | 0.038 | -0.12 | 0.123 | 12 | 0.020 |
| LOG AST [U/l] | 0.02 | 0.785 | 30 | 0.050 | -0.04 | 0.603 | 27 | 0.045 |
| LOG hsCRP [µg/ml] | -0.14 | 0.077 | 11 | 0.018 | -0.18 | 0.020 | 9 | 0.015 |
| LOG hsIL-6 [pg/ml] | -0.10 | 0.186 | 16 | 0.027 | -0.09 | 0.239 | 19 | 0.032 |
| BMI [kg/m^2^] | **-0.26** | **<0.001** | **1** | **0.002** | **-0.22** | **0.004** | **5** | **0.008** |
| Age [year] | -0.03 | 0.671 | 28 | 0.047 | 0.08 | 0.310 | 22 | 0.037 |
| LOG tTestosterone [ng/ml] | -0.18 | 0.023 | 10 | 0.023 | **-0.23** | **0.003** | **4** | **0.007** |
| LOG E2 [pg/ml] | **0.20** | **0.009** | **7** | **0.012** | **0.24** | **0.002** | **3** | **0.005** |

^1^ Ranked from the lowest to highest *p*-value.

^2^ Benjamini-Hochberg correction for multiple testing, with Q set to 0.05.

**III PRINCIPAL COMPONENT ANALYSIS - DATA REDUCTION**

Principal component analysis was used to summarize the inter-related physiological markers of health. PCA returned four PCs with eigenvalues > 1 (Table S3). PC1 was loaded mainly by glucose homeostasis markers (HOMA-IR, C-peptide). PC2 was loaded mainly by lipid profile, mainly markers negatively predicting health (total cholesterol, LDL and triglycerides level). PC3 was loaded mainly by liver enzymes (ALT, AST). PC4 was loaded mainly by inflammatory markers (hsCRP, Il-6). The four PCs jointly explained 69.82 % of the variation in health markers variables.

Table S3. Descriptive statistics and factor loadings for health markers principal components analysis (N=161).

| Eigenvalue  % variance explained | PC1 | PC2 | PC3 | PC4 |
| --- | --- | --- | --- | --- |
|  | 2.89 | 1.88 | 1.57 | 1.34 |
|  | 26.24 | 17.13 | 14.24 | 12.21 |
| LOG Total Cholesterol [mg/dl] | -0.01 | 0.98 | 0.01 | -0.10 |
| LOG HDL [mg/dl] | -0.59 | 0.21 | 0.05 | -0.44 |
| LOG LDL [mg/dl] | 0.16 | 0.89 | -0.04 | 0.16 |
| LOG Triglycerides [mg/dl] | 0.60 | 0.45 | -0.01 | 0.13 |
| LOG HbA1C [%] | 0.16 | 0.12 | -0.14 | 0.25 |
| LOG HOMA-IR | 0.87 | 0.13 | 0.10 | -0.03 |
| LOG C-peptide [ng/ml] | 0.91 | 0.04 | 0.11 | -0.04 |
| LOG ALT [U/l] | 0.21 | 0.04 | 0.88 | 0.12 |
| LOG AST [U/l] | -0.04 | -0.06 | 0.91 | -0.08 |
| LOG hsCRP [µg/ml] | 0.07 | 0.13 | 0.14 | 0.75 |
| LOG hsIL-6 [pg/ml] | -0.08 | -0.09 | -0.02 | 0.78 |
